# Simulated viewing distance impairs the confidence–accuracy relationship for long, but not moderate distances: support for a model incorporating the role of feature ambiguity

**DOI:** 10.1186/s41235-022-00406-5

**Published:** 2022-06-28

**Authors:** Sara D. Davis, Daniel J. Peterson

**Affiliations:** 1 Department of Psychology, University of North Florida, Building 51, Room 3404, Jacksonville, FL 32224 USA; 2 Department of Psychology, Skidmore College, Tisch Learning Center Room, 139, 815 N Broadway, Saratoga Springs, NY 12866 USA

**Keywords:** Eyewitness identification, Face recognition, Viewing distance, Confidence–accuracy relationship

## Abstract

There is an increasing need in eyewitness identification research to identify factors that not only influence identification accuracy but may also impact the confidence–accuracy (CA) relationship. One such variable that has a notable impact on memory for faces is viewing distance, with faces encoded from a shorter distance remembered better than faces encoded at longer differences. In four pre-registered experiments, using both laboratory and online samples, we compared faces viewed at a simulated viewing distance at two different levels (medium and far) to faces that were viewed at a very near-simulated distance. Distance was simulated using a Gaussian blur function with higher levels of blur corresponding to greater simulated distance. We found that both medium and far simulated distances impaired memory performance overall relative to no simulated distance, with increased distance resulting in poorer memory. However, only far simulated distances impaired the CA relationship. In a fourth experiment, we found that a pre-test warning did not ameliorate this impairment of the CA relationship for faces viewed at a far simulated distance. These findings suggest that even high-confidence identifications made for faces viewed from long distances should be disregarded, and that estimator variables that impact memory may degrade the CA relationship when memory is reduced to critical levels.

**Supplementary Information:**

The online version contains supplementary material available at 10.1186/s41235-022-00406-5.

## Significance statement

When an eyewitness views a crime unfold, several variables influence the likelihood that they will be able to later make an accurate identification. Past research claimed that the relationship between confidence and accuracy in these identifications was tenuous at best, but a more recent analysis strategy has changed that perception. In fact, eyewitness confidence may be a good indicator of accuracy so long as the circumstances of the encoding event are ideal. What is less well-known is whether the relationship between confidence and accuracy may be preserved even in non-ideal circumstances. Although it is well established that eyewitness’ memory for faces is degraded with increasing distance, whether this factor impacts the relationship between witness confidence and accuracy is less well-understood. Some studies have found that the relationship between confidence and accuracy may be conserved at some distances but not others. Researchers have suggested that this is driven by eyewitnesses’ failure to appreciate how much more difficult the task of identifying a face is as viewing distance increases. In four experiments, we employed a within-subjects design which eliminates the possibility that individuals can change their strategy to become more or less conservative but does cue them in to the fact that recognition will be harder for some faces than others. We found evidence that only long distances impaired the confidence–accuracy relationship. Moderate distances did not, suggesting that feature ambiguity at encoding leads to the threshold effect rather than global overconfidence in recognition performance.

Due to a relatively recent change in the way that eyewitness memory researchers quantitatively characterize the relationship between confidence and accuracy, there has been a revived interest in identifying factors that do and do not influence the ability of an eyewitness’ confidence in their identification to predict their likelihood of accuracy (Wixted & Wells, 2017). As such, we are interested in the way that certain estimator variables, that is, variables that affect identification accuracy but are *not* under control of the criminal justice system (Wells, 1978), influence this relationship. Whereas a great deal of research in the field of psychology and law focuses rightly on system variables, or those factors that *are* under control of the criminal justice system, estimator variables are just as important to understand (Semmler et al., 2018). That is because we can generate evidence-based predictions of how likely a particular witness is to successfully identify a perpetrator given their specific eyewitness context if we understand the impact of that context on memory performance. For the present studies, we focused on one estimator variable, viewing distance (the distance between the observer and the target face at encoding), to better understand how it influences the relationship between witness confidence and accuracy.

It should come as no surprise to a layperson that as the distance between an eyewitness and the face they need to later identify increases, identification accuracy decreases. The science is clear on this fact (e.g., Lindsay et al., 2008; Nyman et al., 2019; Lockamyier et al., 2020), and this finding has been recognized by official policymakers. For example, Lindsay et al. approached students on a college campus and asked them to encode the face of a research assistant who appeared between 4 and 15 m away or 20–50 m away. Participants then made a lineup decision from a target-absent or target-present lineup immediately or after a 24-h delay. Confirming the intuitive prediction, correct target identification was poorer at the longer distances. In a similar vein, Nyman et al. had participants encode in a more controlled (but still naturalistic) setting at four different distances ranging from 5 to 110 m. Participants similarly made an identification from a lineup, and the same pattern of results emerged. However, the real-world nature of the testing conditions used in both Lindsay et al. (2008) and Nyman et al. (2019) introduces higher variability than what would be ideal by laboratory standards. These studies offer an excellent illustration of the trade-offs that researchers must make between simulating natural conditions closely and exerting rigorous experimental control. While there is no right answer as to which approach is best, other researchers have attempted to simulate viewing distance in the laboratory using digital facsimiles of faces (rather than real, live, faces) corresponding to some physical distance in the natural world. Fortunately, distance can be simulated in several empirically established manners. The first is to record the witnessed event on video and simply vary the distance between the target and the camera (Lockamyier et al., 2020). Researchers may alternatively opt to present participants with photographs of faces but reduce their size to approximate greater distance (e.g., Loftus & Harley, 2005). However, both approaches translate poorly to remote data collection given the lack of standardization with respect to the display size that remote participants opt to use. To address this issue, we chose to blur faces to a greater or lesser extent translating to greater or lesser simulated distance. Face blurring can be accomplished in multiple ways (e.g., Gaussian blur, Lampinen et al., 2015; low-pass filters, Loftus & Harley, 2005). Similar to the logic behind showing images of reduced size, blurring the image of a face simulates the fact that distant faces are represented by fewer photoreceptors in the retina, meaning that each feature of the face must be encoded by fewer cells, resulting in something similar to a pixelated image.

Loftus and Harley (2005) employed such an approach and found that using a low-pass filter perfectly simulated in vivo changes in viewing distance (comparable to reducing photograph size). Following this study, Lampinen et al. (2015) used Gaussian blur (which produces a similar low-pass visual effect) and found evidence consistent with more naturalistic research procedures—a negative monotonic trend for accuracy as simulated distance increased. Therefore, the use of a blur function to simulate distance appears effective and allows researchers to maintain tight laboratory control. This method has an advantage over size manipulations because participants can adjust the visual angle of their screen to ensure that they encode the blurred face as intended, but this does not ameliorate the intended blur effect.

Such a blurring procedure allowed us to investigate how (if at all) distance impacts the relationship between confidence and accuracy in eyewitness memory (Lampinen et al., 2014). Eyewitness misidentification is the single largest contributor to cases that have been overturned in the last several decades by the Innocence Project (Innocence Project, 2019). Accordingly, it is important for researchers to identify the circumstances under which misidentifications are more likely. Confidence judgments commonly accompany witness identifications and may be prompted (“How sure are you that this was the guy?”) or unprompted (“That was for sure the guy I saw!”). Given the frequency with which such information is tied to identifications, it behooves the criminal justice system to determine their usefulness for triers of fact (Smalarz et al., 2021; Wells et al, 1981, 2002). Not even thirty years ago, eyewitness memory researchers were generally in agreement that eyewitness confidence was, at best, weakly related to eyewitness accuracy (Sporer et al., 1995). That sentiment, however, has since shifted; Wixted and Wells (2017) recently made the case in favor of using confidence as a predictor of accuracy in legal contexts, arguing that previous suppositions that confidence and accuracy were unrelated were based on the incorrect analytical approach (i.e., the point-biserial correlation, see Juslin et al., 1996 for a discussion).

Their proposal did come with a caveat: that confidence should only be used as a predictor of accuracy as long as the conditions at the time of encoding and identification of the face were favorable to a positive identification (e.g., estimator variables at the time of encoding are associated with better memory performance, and system-level factors known to influence eyewitness accuracy or confidence are absent). However, some have further argued that these favorable conditions may be a sufficient, but not necessary, condition for confidence to be a valid and reliable predictor of accuracy (Mickes et al., 2017). In essence, there may be several suboptimal conditions that impair eyewitness memory overall but leave the confidence–accuracy relationship intact (see Wixted et al., 2016, for a field study confirming this prediction). This bodes well for the use of confidence as probative evidence in legal contexts, as it provides a filtering mechanism whereby the testimony of low confidence witnesses can be prevented from proceeding to trial (because their low confidence suggests a low likelihood of an accurate identification).

In the context of viewing distance, relatively few studies have attempted to examine the confidence–accuracy relationship specifically. Semmler et al. (2018) re-analyzed the confidence accuracy-relationship from Lindsay et al. (2008) by constructing calibration curves and found that even though viewing distance had large deleterious effects on memory (as indexed by d prime, a measure of discriminability), the confidence–accuracy *relationship* itself remained largely intact (although note that this was true only when the identification was delayed; the same was not true when the identification was immediate). They posed the argument that simply because a given estimator variable affects memory does not mean that the variable will necessarily influence confidence judgments, and that identifications made by highly confident witnesses are still likely to be accurate. Under a global memory and metamemory impairment framework, any variable that has a negative effect on memory overall would also reduce the threshold that an eyewitness might have for making a high confidence judgment. For example, if one were to witness a burglar late at night under poor viewing conditions, we would expect that the likelihood of correctly identifying the burglar later to be quite low. A global framework would predict that on average, calibration would also be impaired and an eyewitness in this scenario (compared to one witnessing under ideal viewing conditions) would be more likely to erroneously assign a higher-confidence judgment to a misidentification, making the confidence judgment an *overestimator* of performance.

What Semmler et al. (2018) and we (Davis et al., 2019) have proposed is that factors that affect memory performance overall do not necessarily impair metamemory judgments (e.g., Connor et al., 1997; Leippe, 1980; Penrod & Cutler, 1995). In essence, the idea is that eyewitnesses know implicitly or explicitly that their memory performance is poor and scale their confidence ratings downward as they make identifications. This leaves the confidence–accuracy relationship largely intact, meaning that triers of fact can still place their faith in high-confidence eyewitness judgments even when the circumstances surrounding the identification are suboptimal. In contrast, Nyman et al. (2019) analyzed the confidence–accuracy relationship in their study using an in vivo testing procedure in a science center and found that the confidence–accuracy relationship appeared to be preserved for shorter distances, but *not* longer distances. Lockamyier et al. (2020) identified a similar pattern in their confidence–accuracy characteristic curves, with good calibration observed at short distances (3 m) but not at longer distances (10 and 20 m), again showing that increasing distance may be associated with impairments to the confidence–accuracy relationship. Similar findings have also been demonstrated for estimator variables other than viewing distance (e.g., the cross-race effect; Dodson & Dobolyi, 2016).

According to Nyman et al.’s (2019) threshold model, participants in conditions where discriminability is especially low may fail to realize just how difficult the task is, and that success is unlikely. This leads them to approach making their metacognitive judgments in the way that they would for only a moderately difficult task, which means that the majority of their judgments will overestimate their actual performance. Research on metacognition in the education literature supports this idea; learners tend to be more overconfident for very difficult material compared to moderately difficult material, because they tend to erroneously assume that their level of performance will remain the same even as difficulty increases (e.g., Kelemen et al., 2000; Maki et al., 2005; Schraw & Roedel, 1994).

However, both Nyman et al. (2019) and Lockamyier et al. (2020) acknowledged that there were relatively few positive identifications made at the very long distances, making it difficult to calculate the appropriate inferential statistics at these highest levels of confidence and casting into doubt whether the threshold effect observed was simply due to high variability in rare high-confidence judgments at long distances. This methodological problem is exacerbated by the lineup procedure, which has high applied value but by necessity only yields a single data point per condition, further increasing variability. In the present studies, we examined the impact of viewing distance on the confidence–accuracy relationship using a face recognition paradigm, which generates dozens of observations per condition. This in turn yields more reliable estimates of accuracy at each level of confidence. Whereas single-face lineup identifications and old/new decisions for a large set of previously presented faces are not superficially identical tasks, they both rely on similar cognitive architectures of facial processing (Morgan et al., 2007), with face recognition often being useful as a basic research tool for testing theoretical positions. Although there are important differences between face recognition and lineup paradigms (but see Weber & Brewer, 2006, for a discussion of the similarities between the paradigms), we believe that the increased power and stability of the estimates outweigh the disadvantages of reduced ecological validity.

In addition to maximizing statistical power by using a face-recognition paradigm, we conducted the current studies in a more powerful within-subjects design meant to maximize the reliability of point estimates. Whereas Nyman et al. (2019) did require participants to make four lineup identifications of possible targets, these were subdivided into both target-present and target-absent lineups, and each task was completed sequentially (e.g., participants studied and were tested on a single face at a time), making it more likely that participants could adjust their global criteria for choosing and for assigning confidence judgments from task to task. We argue that while the data provided by Nyman et al. provide some evidence in favor of a threshold account, more rigorous laboratory testing should be done with a sample and design created to detect fluctuations in the nature of the confidence–accuracy relationship.

We also conducted our studies using both college students and workers on Amazon’s Mechanical Turk (MTurk). Because eyewitnesses in the USA can probabilistically be any of a number of demographic profiles present in the country, it is important that participant samples match this level of diversity. Thus, the MTurk sample allows a more general estimate of the abilities of the US population,^1^ rather than just relying on a highly educated, homogenous sample of college students to generalize to the population of interest. Finally, we implemented a simple procedural tactic (i.e., an instructional warning) meant to correct overestimates of confidence in Experiment 3, as it is important to determine whether any negative influence of estimator variables can be corrected by policy changes that can easily be implemented in the field.

## Experiments 1a and 1b

Experiments 1a-3 were pre-registered at the Open Science Framework. These pre-registrations, Supplementary Materials, and all materials and data used for the analyses here are available at https://osf.io/7wdvy/.

### Method

#### Participants

We determined sample size based on our previous research (Davis et al., 2019), in which we found that a sample of 50 participants per condition was sufficient to examine differences between conditions in calibration curves. Participants for Experiment 1a and 1b were 102 students attending Skidmore College who participated for partial course credit (*M*_Age_ = 19 years), with 51 participants in each experiment.

#### Materials and procedure

**Fig. 1:**
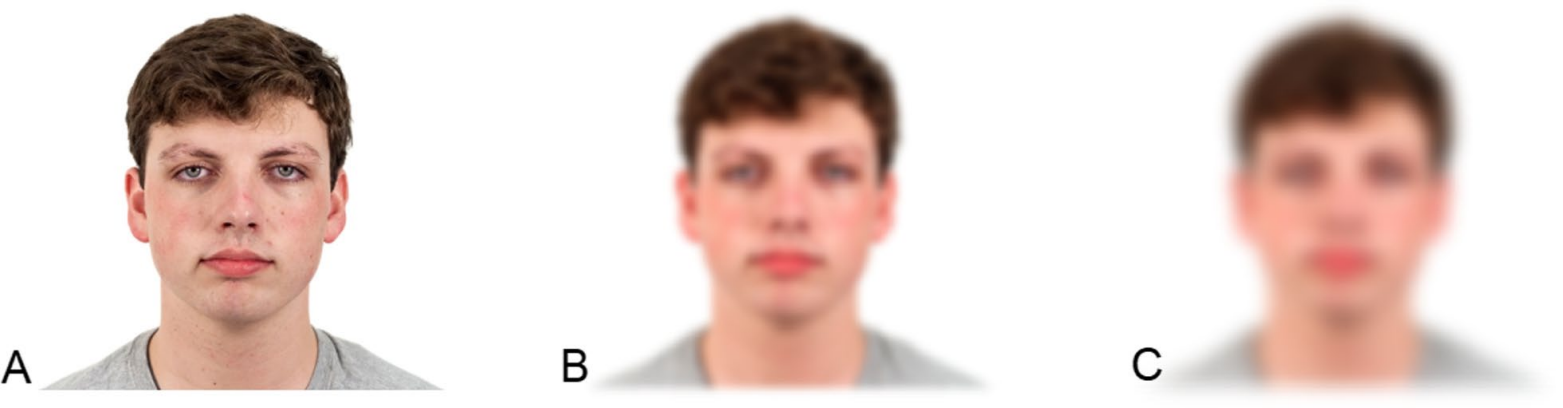
Example Stimuli for Experiments 1–3.* Note: *Panel **A** depicts a clear face, Panel **B** depicts the medium simulated distance (Gaussian Blur Level 5, Experiments 1a, 2, and 3), and Panel **C** depicts the far simulated distance (Gaussian Blur Level 10, Experiments 1b, 2, and 3).

The procedure was similar to that of Davis et al.’s (2019) Experiment 2. The stimuli were 60 male and female faces of different races (e.g., Caucasian, Hispanic, African-American, and Asian) with a neutral close-mouthed expression from the Chicago Face Database (Ma et al., 2015). Distance was simulated (see Fig. 1 for example stimuli) by applying a Gaussian blur of 5 for Experiment 1a and a blur of 10 for Experiment 1b over the photographs using Sketch photograph editing software. Gaussian blur is a method of photograph smoothing which creates an average color value for each pixel based on the other pixels surrounding it, weighting closer pixels more heavily. The level of blur refers to the number of surrounding pixels considered in the calculation; for a Gaussian blur of 5 (for Experiment 1a), each pixel would contain the Gaussian-weighted average of the five pixels surrounding it in each direction. In Experiment 1b, the blurred faces used a Gaussian blur factor of 10, which averages across the nearest 10 pixels and thus produces a blurrier image. We estimate that the Gaussian blurs of 5 and 10 correspond to approximately 43 and 172 feet (see Loftus & Harley, 2005; but note that the Gaussian blur filter used here is slightly different from their low-pass filter). Each participant encoded 30 faces, 15 of which were clear and 15 of which were blurred. We will refer to the clear condition as the near-distance condition and the blurred conditions as the medium- (Experiment 1a) or far- (Experiment 1b) distance conditions. The presentation order of the near and simulated distant faces was randomized separately for each participant, and assignment of a given face to be studied or not and near or distant was counterbalanced across participants.

Prior to encoding, participants were told that they would see a series of faces, some blurred and some unblurred, and that their memory for these faces would be tested later. They then encoded each face for 1.5 s. Following the encoding phase, participants completed a series of word puzzles for 2 min and then received instructions for the recognition test. During the test, all 60 faces (30 that were previously encoded, and 30 that were new) were presented without blur, much like when a witness views faces in a lineup. Faces were presented one at a time in a random order, and participants were first asked to indicate whether a given face was seen or not seen, and then to indicate their confidence in their response on a scale from 0 (a guess) to 100 (certain). Both the old/new recognition task and the confidence judgments were self-paced. After completing the test, participants answered a series of demographic questions and were debriefed.

### Results

#### Experiment 1a

We first examine the impact of simulated viewing distance on memory performance overall (see Table 1 for a summary of hits, false alarms, and d prime scores). D prime scores represent a standardized measure that deducts false alarms^2^ (incorrectly responding “Seen” to a new face) from hits (correctly responding “Seen” to an old face), with scores of zero indicating no discriminability between old and new faces and higher scores representing better levels of discriminability. Unsurprisingly, discriminability was much poorer for faces that had been encoded at a medium simulated distance (*M* = 0.99) than faces that had been encoded at a near simulated distance (*M* = 1.77), *t*(50) = 6.14, *p* < 0.001, *d* = 0.86.

**Table 1:** Hits, false alarms, and d' values for each condition in experiments 1–3

	Hits	False alarms	*d'*
*Experiment 1a*			
Near	0.77 *(0.13)*	0.21 *(0.15)*	1.77 *(0.76)*
Medium distance	0.52 *(0.20)*	0.21 *(0.17)*	0.99 *(0.74)*
*Experiment 1b*			
Near	0.79 *(0.13)*	0.23 *(0.15)*	1.77 *(0.71)*
Far distance	0.40 *(0.14)*	0.22 *(0.11)*	0.58 *(0.44)*
*Experiment 2*			
Near	0.69 *(0.20)*	0.32 *(0.22)*	1.17 *(0.87)*
Medium distance	0.54 *(0.20)*	0.37 *(0.24)*	0.54 *(0.67)*
Near	0.63 *(0.21)*	0.33 *(0.20)*	0.92 *(0.83)*
Far distance	0.38 *(0.20)*	0.29 *(0.19)*	0.29 *(0.80)*
*Experiment 3*			
No warning			
Near	0.76 *(0.15)*	0.36 *(0.23)*	1.30 *(0.90)*
Far distance	0.46 *(0.23)*	0.37 *(0.24)*	0.32 *(0.52)*
Warning			
Near	0.68 *(0.20)*	0.29 *(0.21)*	1.23 *(0.88)*
Far distance	0.41 *(0.20)*	0.30 *(0.23)*	0.41 *(0.64)*

SD are in parentheses.

**Fig. 2:**
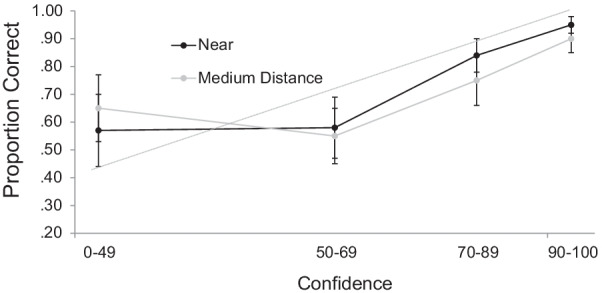
Calibration Curves for Faces that were Presented at a Near or Simulated Medium Distance in Experiment 1a *Note:* The diagonal line indicates optimal calibration. Bars represent descriptive 95% confidence intervals

Of critical interest was the calibration between confidence and accuracy. Given the greater applied and theoretical importance of old relative to new responses, we only analyzed data for faces which participants identified as old (see Weber & Brewer, 2003 for a discussion of why old responses are more important for understanding the confidence–accuracy relationship in eyewitness memory). We divided old responses into four confidence bins, whose size was based on our prior research (see Davis et al., 2019 for a discussion of this method), and plotted overall accuracy (e.g., the number of times a participant correctly responded old divided by the total number of old responses) as a function of each of these four levels of confidence.^3^ As can be seen in Fig. 2, calibration was generally quite good for faces that were encoded under both the.

near- and medium-distance conditions. Four paired-samples *t* tests confirmed that there was no significant difference between the two conditions at any of the four levels of confidence, *t*s < 1.69. Thus, whereas memory was impaired overall when faces were encoded at a medium distance, there was no impact on the *relationship* between confidence and accuracy. This is largely consistent with the extant literature that has examined the impact of estimator variables on the confidence–accuracy relationship (e.g., Davis et al., Palmer et al., 2013).

#### Experiment 1b

**Fig. 3:**
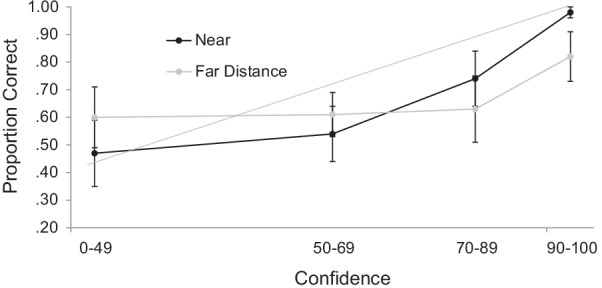
Calibration Curves for Faces that were Presented at a Near or Simulated Far Distance in Experiment 1b. *Note:* The diagonal line indicates optimal calibration. Bars represent descriptive 95% confidence intervals.

Again, we first examined memory accuracy between the clear and simulated distant faces using d prime as the dependent measure (see Table 1). As in Experiment 1a, discriminability was lower for faces that were presented at a far simulated distance (*M* = 0.58) than when they were presented clearly to simulate a near distance (*M* = 1.77), *t*(50) = 11.42, *p* < 0.001, *d* = 1.60.

Once more, we were primarily interested in the relationship between confidence and accuracy, depicted in Fig. 3. Here, there were no differences between conditions at the first three levels of confidence, *t’*s < 1.41, but accuracy was significantly worse for faces encoded at a far simulated distance relative to faces that were encoded at the near simulated distance at the highest bin of confidence, *t*(35) = 3.39, *p* = 0.002, *d* = 0.57.^4^

## Discussion

Experiments 1a and 1b revealed a seemingly disparate pair of findings similar to Lockamyier et al. (2020) and Nyman et al. (2019). Whereas encoding at a simulated distance did impair memory overall in both experiments, the impact of simulated viewing distance on the confidence–accuracy relationship depended on the level of distance. In Experiment 1a confidence was predictive of accuracy across all four confidence bins. However, at a longer simulated distance, participants were less accurate for faces that had been encoded at a far simulated distance than the near distance. This finding is particularly problematic for triers of fact in the criminal justice system, as high-confidence identifications are the most likely to serve as probative evidence at trial. Thus, if confidence is not a predictor of accuracy at high levels of confidence for faces viewed at a far distance, recommendations for best practices under these conditions would likely include not allowing such eyewitnesses to testify at all. Therefore, we thought it prudent to replicate this finding using a different sample in a single experiment (see Open Science Collaboration, 2015; for a discussion of the importance of reproducibility in psychological science). Specifically, we compared the difference between the near-, medium- and far-distance conditions in a single experiment using workers on Amazon’s Mechanical Turk.

## Experiment 2

### Method

#### Participants, design, materials, and procedure

One-hundred and five MTurk workers participated for monetary compensation (*M*_Age_ = 40 years), with 53 participants in near-distance condition and 52 participants in the far-distance condition. The materials used here were identical to those used in Experiments 1a and 1b. However, we manipulated the degree of Gaussian blur (medium-distance and far-distance) between subjects in a single experiment. Thus, the design was a 2 (Face Distance: Near vs. Distant) × 2 (Level of Distance: Medium vs. Far) mixed design, with Face Distance manipulated within-subjects and Level of Distance manipulated between-subjects.

## Results and discussion

We first examined memory performance overall with a 2 (Face Distance: Near vs. Distant) × 2 (Level of Distance: Medium or Far) mixed ANOVA (see Table 1). There was a main effect of Face Distance, *F*(1, 103) = 50.69, *p* < 0.001, η_p_^2^ = 0.33, with lower discriminability for faces that were encoded at a simulated distance. There was also a nearly significant main effect of Level of Distance, *F*(1, 103) = 3.97, *p* = 0.05, η_p_^2^ = 0.04, with lower discriminability in the Far Distance condition than the Medium Distance condition. The interaction was not significant, *F*(1, 103) < 0.001, *p* = 0.99, η_p_^2^ < 0.001. Thus, memory performance was worse for distant faces than near faces and was worse still for the far distance relative to the medium distance.

**Fig. 4:**
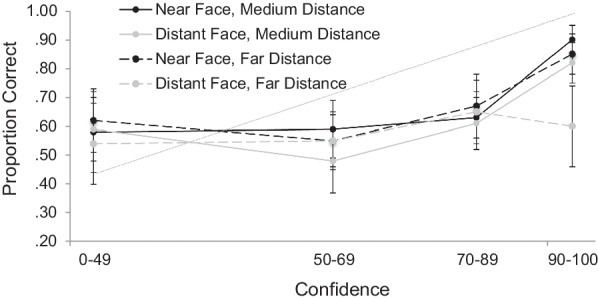
Calibration Curves for Faces that were Presented at a Near or Simulated Distance (Medium or Far) in Experiment 2. *Note:* The diagonal line indicates optimal calibration. Bars represent descriptive 95% confidence intervals

As in Experiments 1a and 1b, we were primarily interested in the impact of the varying levels of distance on the relationship between confidence and accuracy (see Fig. 4). As specified in our pre-registration, we conducted four separate 2 (Face Distance: Near vs. Distant) × 2 (Level of Distance: Medium or Far) mixed ANOVAs at each level of confidence. At the first three levels of confidence, there were no main effects or interactions, *F*’s < 1.79. At the highest level of confidence, there was a main effect of Face Distance, *F*(1, 67) = 21.39, *p* < 0.001, η_p_^2^ = 0.24, as well as a main effect of Level of Distance, *F*(1, 67) = 8.08, *p* = 0.006, η_p_^2^ = 0.11. These main effects were qualified by a significant interaction, *F*(1, 67) = 4.90, *p* = 0.03, η_p_^2^ = 0.07. Follow-up tests at this highest level of confidence (using a Bonferroni correction for multiple comparisons, critical alpha = 0.013) revealed that in the medium-distance condition, the comparison between the near and distant faces approached but did not reach significance, *t*(39) = 2.41, *p* = 0.02, *d* = 0.38. In the far-distance condition, the comparison between near and distant faces was significant, *t*(28) = 3.59, *p* = 0.001, *d* = 0.67, with lower accuracy for the faces that were encoded at a far distance (*M* = 0.60) than for faces that were encoded at a near distance (*M* = 0.85). There was no difference between the medium-distance and far-distance conditions for faces that were encoded at a near distance, *t*(89) = 1.20, *p* = 0.23, *d* = 0.25. However, accuracy was lower for faces that were encoded at a far distance (*M* = 0.60) than faces that were encoded at the medium distance (*M* = 0.82), *t*(70) = 2.98, *p* = 0.004, *d* = 0.71.

Similar to Experiments 1a and 1b, it appears that there was little impact of simulated distance on the confidence–accuracy relationship at the lower level of distance. However, at the greater level of distance, participants were significantly more overconfident relative to clear faces and to faces viewed from a medium distance. The goal of Experiment 3 was to attempt to implement an instructional warning (see Blank & Launay, 2014, for a meta-analysis on similar warning manipulations in the misinformation literature) designed to ameliorate the effect of encoding faces at a far simulated distance on the confidence–accuracy relationship.

## Experiment 3

### Method

#### Participants, design, materials, and procedure

Participants in Experiment 3 were 52 undergraduates at Skidmore College and 54 workers from Amazon’s Mechanical Turk. As can be seen in our pre-registration, we anticipated collecting the entire sample from the population at Skidmore College. However, due to the COVID-19 pandemic, in-person data collection was suspended indefinitely. We elected to complete data collection using an MTurk sample in order to facilitate the timely dissemination of data. Descriptively the MTurk sample was older (*M*_Age_ = 29.26) than the Skidmore sample (*M*_Age_ = 18.67 years) and more likely to identify themselves as male (*M*_MTurk_ = 60%; *M*_Skidmore_ = 32%). Three MTurk participants were eliminated for failing to follow instructions and/or having a false alarm and hit rate of 1.0 (i.e., indicated that every face was seen previously). This yielded 51 MTurk participants in the final sample.

The design was identical to Experiment 1b (i.e., participants encoded both clear faces to simulate a near distance and faces blurred with the highest level of blur to simulate the far distance), with the addition of a warning manipulation. Immediately prior to beginning the recognition test, half of participants were given a warning and half were not. The goal of the warning instruction was to examine whether a brief intervention could reduce the overconfidence effect for faces that were encoded at a long simulated distance. If successful, this type of instruction could easily be implemented in the field prior to lineup administration. The warning read as follows:

It is very important that you consider your confidence judgments carefully. Some of the faces you saw previously were blurred, and this can make recognizing the faces very difficult. So please make sure that you only give very high confidence judgments to faces that you are ABSOLUTELY SURE that you saw in the first phase.

For the Skidmore sample, participants saw a screen immediately prior to the test to retrieve their experimenter, who then delivered the warning verbally. After they had been verbally warned, the following instruction screen repeated the warning in text, with the words “It is very important that you consider your confidence judgments carefully” printed in bold font and the words “ABSOLUTELY SURE” printed in bold and red font. For the MTurk sample, participants saw a pre-recorded video of a research assistant delivering the warning with closed captioning,^5^ and were not able to alter the playback of the video or advance to the next screen until the video had finished playing. Once they had finished the video and the page had advanced, the instructions were repeated in text as in the Skidmore sample. After the test, participants in the MTurk sample were asked to briefly describe the content of the video that they had seen to verify that they had understood the instruction.^6^ Because the Skidmore sample heard the warning from a live research assistant, we did not anticipate the necessity of such a question, and so this question was only asked for the participants from MTurk.

## Results and discussion

We include Sample (Skidmore vs. MTurk) as a factor in each of the analyses reported below. As we had not anticipated collecting data from two different samples, this factor was not a part of our preregistration. However, we adhere to all other aspects of the preregistration, and think it prudent to include this unanticipated factor here as it is most in line with the spirit of open science practices.

We first examined the effect of simulated viewing distance on memory performance overall in a 2 (Sample: Skidmore vs. MTurk) × 2 (Distance: Near vs. Far Distance) × 2 (Warning: Warning vs. No Warning) ANOVA. There was a significant effect of simulated distance, *F*(1, 99) = 131.17, *p* < 0.001, η_p_^2^ = 0.57, with faces that had been encoded at the far distance (*M* = 0.37) remembered more poorly than faces that had been encoded at the simulated near distance (*M* = 1.26). There was also a main effect of sample, *F*(1, 99) = 34.92, *p* < 0.001, η_p_^2^ = 0.26, with Skidmore participants demonstrating better memory overall (*M* = 1.13) than MTurk participants (*M* = 0.49). The main effect of warning was not significant, *F*(1, 99) = 0.01, *p* = 0.95, η_p_^2^ = 0.01. Sample interacted with Distance, *F*(1, 99) = 16.03, *p* < 0.001, η_p_^2^ = 0.14, but all other two-way interactions were not significant, *F*’s < 1.24, and the three-way interaction was only marginal, *F*(1, 99) = 3.19, *p* = 0.08, η_p_^2^ = 0.03. The Sample × Distance interaction signifies that the performance difference between the Skidmore (*M* = 1.72) and MTurk (*M* = 0.79) samples was larger for the near-distance faces, *t*(101) = 6.31, *p* < 0.001, *d* = 1.24, than the far distance faces (*M*_*Skidmore*_ = 0.53, *M*_*MTurk*_ = 0.20), *t*(101) = 2.94, *p* = 0.004, *d* = 0.58. Thus, the results from this analysis suggest that, as before, faces encoded at a far simulated distance were remembered more.

poorly than faces that were encoded at a near simulated distance. Further, MTurkers had poorer memory performance overall, and this performance deficit was reduced when memory performance was reduced for all participants by blurring the faces. While the finding that MTurkers had poorer memory performance is interesting, it is perhaps not surprising that memory performance is worse for older participants who represent a more general swath of the US population than undergraduates at a selective liberal arts college who may have a great deal of experience taking memory tests. However, we would also encourage the reader to consider that the Skidmore sample was collected just prior to the COVID-19 pandemic, whereas the MTurk sample was collected nearly 1.5 years into the pandemic. Thus, the participants may have differed in other critical ways. Importantly, the warning did not influence memory discriminability as indexed by d prime in either sample, although distance again had a large impact on memory performance.

**Fig. 5:**
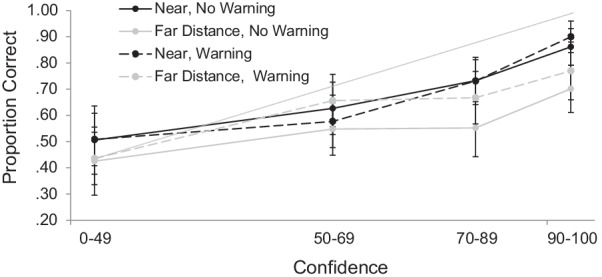
Calibration Curves for Faces that were Presented at a Near or Simulated Far Distance and either Warned or Not Warned in Experiment 3. *Note:* The diagonal line indicates optimal calibration. Bars represent descriptive 95% confidence intervals

As before, our main interest was in the confidence–accuracy relationship (see Fig. 5). At each bin of confidence, we conducted a 2 (Sample: Skidmore vs. MTurk) × 2 (Warning: Warning vs. No Warning) × 2 (Distance: Near vs. Far) ANOVA on conditional accuracy of old responses. There were no significant effects at the first level of confidence, *F*’s < 1. At the second bin of confidence (50–69), there was an interaction between Sample and Distance, *F*(1, 67) = 4.34, *p* = 0.04, η_p_^2^ = 0.06. All other main effects and interactions at this level of confidence were not significant, *F*’s < 1.32. To avoid inflating the likelihood of a Type I error by conducting multiple ANOVAs on these data, and the fact that Sample was not an a priori factor of interest here, we do not decompose this interaction. As confidence increased to the third bin (70–89), the difference between near (*M* = 0.71) and far distance (*M* = 0.61) faces emerged, *F*(1, 76) = 4.35, *p* = 0.04, η_p_^2^ = 0.05, as did a difference between the Skidmore (*M* = 0.76) and MTurk (*M* = 0.56) samples, *F*(1, 76) = 16.35, *p* < 0.001, η_p_^2^ = 0.18. No other effects approached significance at this third level of confidence, *F*’s < 1.62. We last examine the highest level of confidence, which is of important applied interest. Here, the main effect of Sample was significant, *F*(1, 67) = 13.03, *p* < 0.001, η_p_^2^ = 0.16, with MTurk workers (*M* = 0.71) again showing poorer memory performance relative to Skidmore students (*M* = 0.87). As in Experiments 1b and 2, the far distance (*M* = 0.73) was associated with poorer accuracy than the near distance (*M* = 0.84), *F*(1, 67) = 5.97, *p* = 0.02, η_p_^2^ = 0.08, and no other main effects or interactions emerged, *F*’s < 2.20. Thus, whereas inspection of Fig. 5 shows that the warning numerically improved accuracy for the far distance faces, this was not borne out statistically, indicating that the warning was ineffective at improving the confidence–accuracy relationship for faces encoded at a far simulated distance.

## General discussion

In two unique populations and four experiments, we found evidence supporting the idea that while both medium and far simulated distances (relative to a near simulated distance) impair face recognition overall, only far distances impair the confidence–accuracy relationship (see Supplemental Materials for a combined analysis of all experiments). This was particularly true for the highest bins of confidence (90–100%), where participants were consistently more overconfident (i.e., their accuracy was numerically lower than their confidence judgment) in their accuracy for the faces that were encoded at a simulated far distance than simulated medium distance. This latter finding has important applied as well as theoretical value. Eyewitnesses with the highest levels of confidence in their selections are the most likely to proceed to trial, thus having the potential to lead to the conviction of innocent suspects (Garret, 2011). Whereas past research has suggested that this relationship may be impaired with increasing distance (e.g., Lockamyier et al., 2020; Nyman et al., 2019), the studies reported here are the first to do so in a within-subjects face recognition design and with sufficient power to provide stable estimates of the confidence–accuracy relationship at the highest levels of confidence (see the Supplemental Materials for a combined analysis of data from all four experiments supporting this assertion).

In the present studies, we report two additional important findings. In Experiment 3, we attempted to implement a simple instructional warning to reduce the overconfidence associated with the far simulated distance. Such manipulations are common in the field of eyewitness memory (see Blank et al., 2014 for a meta-analysis) and would provide a simple correction for the deleterious effect of increasing distance on the confidence–accuracy relationship. Unfortunately, this instructional warning was not effective. We will return to the applied implications of this finding shortly, but this suggests that eyewitnesses who view a face from a sufficiently far distance do not make good eyewitnesses even if they make identifications with high confidence.

We also found that MTurk workers were less accurate recognizers of faces than our Skidmore sample. This is not particularly surprising, as Skidmore College is a selective liberal-arts college with high admission standards and students that were likely highly motivated to perform well. Students were also tested in-person under the supervision of a research assistant, and students were younger than the MTurk population (see Follmer et al., 2017 for a review of the advantages and disadvantages of using an MTurk sample). Given that we cannot disentangle these many confounding variables contributing to the sample effect, we will not discuss it further. Rather, we simply acknowledge that it appears that MTurk workers completing a face recognition task remotely may not achieve the same levels of accuracy as college students in the laboratory.

Importantly, the results of the present series of studies have implications for our understanding of how estimator variables impact the confidence–accuracy relationship. In particular, the finding that the effects on memory overall and the effects on the confidence–accuracy relationship are separable is theoretically meaningful. This suggests that distance has a quantitative impact on discriminability, and there may be a qualitative threshold at which metacognitive judgments rely less on memory information (which should be quite poor, driving confidence judgments downward) and more on some other internal or external factor, such as the motivation to remember items well. Critically, we observed this pattern when distance was manipulated *within-subjects*. One could certainly make the case that for very difficult recognition tasks in which memory information is sparse, participants may relax their criterion for calling an item “seen” and *also* relax their criterion for what sorts of memory information is required to make a high-confidence judgment (Cox & Dobbins, 2011). This cannot be the case here, because there was not a similar pattern of overconfidence observed for clear faces, and the relatively good memorability of those faces (half of the overall set of old photographs) makes it less likely that participants would be motivated to change their criterion on the recognition task. However, it is important that we include the caveat that, given our experimental design, we cannot rule out the possibility that metacognitive judgments could follow a systematic pattern of degradation rather than a threshold-based pattern. That is, it may be the case that as simulated distance increases from our medium distance to the far distance, accuracy at the highest level of confidence also drops monotonically. Future research that further clarifies the effect of simulated distance on confidence–accuracy calibration at a finer grain of distance manipulations may be a fruitful avenue for future research in this area.

Therefore, we advocate for a modification of the threshold model proposed by Nyman et al. (2019) and supported by Lockamyier et al. (2020). Nyman et al. argued that, under a threshold model, as discriminability decreases to very low levels, eyewitnesses are not able to judge the difficulty of the task and scale their confidence judgments downward accordingly. Following from their data in a lineup task, this is a reasonable position. However, the patterns of data presented in the four studies here are not compatible with this version of the threshold account. If they were, one would expect to observe similar reductions in discriminability and overconfidence for faces encoded at a near simulated distance and at the medium simulated distance. Instead, only faces encoded from a far simulated distance showed reductions in accuracy at the highest levels of confidence.

In a similar line of logic, making participants aware of the difficulty of the task should reduce the overconfidence effect. However, the instructional warning doing so in Experiment 3 was ineffective at weakening the impact of distance on the confidence–accuracy relationship. Thus, we argue for a threshold account that incorporates feature ambiguity. The idea behind feature ambiguity is that when participants encode faces from very far away (but not a moderate distance away), the distance increases the ambiguity in individual facial features. When participants encounter faces that are not at a simulated distance at test, these ambiguous encoded features are more likely to trigger a memory match to both previously seen and not previously seen faces. When this match is erroneous, the participant will nevertheless feel a strong sense of recognition and make an affirmative judgment with high confidence. This is less likely to happen with moderate distances because features retain more of their distinctive characteristics at encoding, reducing erroneous matches to memory of unseen faces.

The applied implications of these experiments are clear. Whereas participant who saw faces at a medium simulated distance were relatively well-calibrated, those who saw faces at the far simulated distance were not, suggesting that eyewitnesses who see a target face from too far away should not be asked to make identifications. Precisely what constitutes “too far away” is likely dependent on the other conditions present at encoding as well as individual differences in memory ability and metacognition, and estimates of viewing distance may be subjective in some cases. Based on the data presented here, we argue that identifications made by witnesses who viewed a perpetrator at very long distances should at the very least be heavily scrutinized.

It is important to note, though, that the procedure in the present experiments involved presenting participants with the same image at encoding (albeit in some conditions blurred) and at retrieval, and simulated distance via a blurring function rather than by manipulating physical distance, which differs from the task typically asked of eyewitnesses in the field. Further, the face recognition paradigm wherein a participant views many faces and must recognize them again from a larger pool is a different task than seeing a single suspect commit a crime and later identifying them from a lineup. We must acknowledge that some aspects of this paradigm which allow for more precise statistical estimates and analysis may place the experiments well outside their intended naturalistic context (see Kovera & Evelo, 2021; Hyman, 2021 for recent discussions of this issue). However, we believe that the findings of the present study do have important implications for how eyewitnesses make recognition judgments for distantly viewed faces even though overall memory accuracy may differ somewhat when placed in these real-world scenarios.

## Conclusions

In four pre-registered experiments across two separate US samples, we found that the confidence–accuracy relationship was preserved for faces encoded at a moderate simulated distance but was impaired for faces encoded at a far simulated distance. This finding supports a threshold model incorporating feature ambiguity, which proposes that as features become more distant, the likelihood of a perceived match to memory at test for new faces becomes more likely, skewing confidence judgments upward. This finding also has important ramifications for how eyewitness identifications made after encoding at a distance are interpreted by the criminal justice system. We argue that in the case of distance, triers of fact be extremely cautious in using confidence judgments as predictors of accuracy.

## Open practices statement

All data required to conduct the analyses reported here as well as any materials are available for download on the Open Science Framework at https://osf.io/7wdvy/. All of the experiments reported here were preregistered at the Open Science Framework as well, and those preregistrations may be found at the link above.

## Supplementary Information

**Additional file 1**

## Data Availability

The datasets analyzed during the current studies as well as Supplementary Materials are available in the Open Science Framework repository, [https://osf.io/7wdvy/].

## Footnotes

1 Keep in mind that the Nyman et al. (2019) sample was a community-based sample at a Science Center, whereas the Lockamyier (2020) sample was the typical college student sample.

2 Note that 30 new faces were presented at test. We counterbalanced assignment of half of these faces to the near condition and half to the medium distance condition for the purposes of statistical analysis. However, there was no functional difference between near and distant new faces as the faces were always presented without blur at test.

3 See Table S1 for the total number of observations analyzed at each bin of confidence.

4 Note that participants who did not have data for both the clear and simulated distance condition at each level of confidence were not included in the statistical test. Thus, the degrees of freedom at each bin of confidence are lower than in the d prime analysis.

5 We included closed-captioning for the warning video in the event that a participant did not have their volume enabled and to increase accessibility. This maximized the likelihood that participants would be exposed to the warning instruction in the remote format.

6 Because this question was open-ended and did not include language specifically querying the content of the warning, it did not serve as a fruitful way to identify participants who had not encoded the warning.
