# Supplementary material for: Simulated viewing distance impairs the confidence–accuracy relationship for long, but not moderate distances: support for a model incorporating the role of feature ambiguity
Source: Cogn Res Princ Implic. 2022 Jun 28;7:55. doi: 10.1186/s41235-022-00406-5 (PMC9240149; doi:10.1186/s41235-022-00406-5)
Supplement: Supplementary file 1 — Additional file 1 [file 41235_2022_406_MOESM1_ESM.pdf]

Supplemental Materials

Table S1

Number of Hits/False Alarms/Participants in Each Condition for the Confidence-Accuracy Calibration Analysis in Experiments 1a, 1b, 2, and 3.

	Number of hits	Number of false alarms	Number of participants
**Experiment 1a (*N* = 51)**			
Near Bin 1	61	50	34
Near Bin 2	84	65	45
Near Bin 3	120	28	44
Near Bin 4	325	16	51
Medium Distance Bin 1	63	36	33
Medium Distance Bin 2	92	69	46
Medium Distance Bin 3	90	33	44
Medium Distance Bin 4	150	21	45
**Experiment 1b (*N* = 51)**			
Near Bin 1	55	65	44
Near Bin 2	81	59	48
Near Bin 3	88	37	43
Near Bin 4	384	14	51
Far Distance Bin 1	57	43	34
Far Distance Bin 2	107	72	46
Far Distance Bin 3	70	32	42
Far Distance Bin 4	73	21	36
**Experiment 2 (*N* = 105)**			
Near Face, Medium Distance Bin 1	74	45	30
Near Face, Medium Distance Bin 2	106	79	44
Near Face, Medium Distance Bin 3	118	84	47
Near Face, Medium Distance Bin 4	253	44	48
Near Face, Far Distance Bin 1	97	61	38
Near Face, Far Distance Bin 2	83	80	43
Near Face, Far Distance Bin 3	106	56	42
Near Face, Far Distance Bin 4	202	61	43
Distant Face, Medium Distance Bin 1	74	66	30
Distant Face, Medium Distance Bin 2	81	88	40
Distant Face, Medium Distance Bin 3	116	82	48
Distant Face, Medium Distance Bin 4	159	54	41
Distant Face, Far Distance Bin 1	50	51	31
Distant Face, Far Distance Bin 2	89	74	43
Distant Face, Far Distance Bin 3	73	35	40

Distant Face, Far Distance Bin 4	81	65	31
**Experiment 3 (*N* = 103)**			
Near, No Warning Bin 1	57	63	34
Near, No Warning Bin 2	109	68	38
Near, No Warning Bin 3	109	57	39
Near, No Warning Bin 4	273	60	46
Near, Warning Bin 1	82	88	39
Near, Warning Bin 2	90	62	43
Near, Warning Bin 3	136	62	50
Near, Warning Bin 4	254	26	48
Far, No Warning Bin 1	50	48	29
Far, No Warning Bin 2	99	83	38
Far, No Warning Bin 3	78	65	35
Far, No Warning Bin 4	108	68	38
Far, Warning Bin 1	88	88	36
Far, Warning Bin 2	85	60	40
Far, Warning Bin 3	98	67	47
Far, Warning Bin 4	68	31	35

Note: Number of participants refers to participants with at least one hit or false alarm in each condition who contributed to the analysis for that condition.

Combined Analysis

To further clarify the point estimates associated with each distance condition, we combined the data from Experiments 1a, 1b, 2, and 3 together in a single analysis. While we recommend that researchers interpret such cross-experimental comparisons with caution, we think that this approach can serve some applied value here. This approach allows us to compare performance between our two conditions quantitatively across two different samples and time periods, substantially increasing power relative to any one experiment. We included both the warned and not warned conditions from Experiment 3, as these conditions did not differ statistically from each other (although recall that the warning numerically increased accuracy at the highest level of confidence). In the interest of transparency, we also note here that this analysis is exploratory, and was not part of our preregistered analytic plan. We conducted a 2

(Level of Distance: Medium or Far) × 2 (Sample: Skidmore or MTurk) ANOVA on accuracy at the highest level of confidence only for the simulated distant faces. Here, there was a main effect of Distance, $F(1, 222) = 12.36$, $p < .001$, $\eta_p^2 = .05$, and a main effect of Sample, $F(1, 222) = 9.02$, $p = .003$, $\eta_p^2 = .04$, but no interaction $F(1, 222) = 0.64$, $p = .43$, $\eta_p^2 = .003$. Accuracy was higher for the faces that were encoded at a medium simulated distance ($M = .86$) than the faces encoded at a far simulated distance ($M = .72$), and Skidmore college participants ($M = .85$) were more accurate than MTurk workers ($M = .73$). Thus, this analysis confirms the pattern noted across Experiments 1a-3, that higher simulated distances impaired the confidence-accuracy relationship but that moderate simulated distances had a minimal impact on the relationship. We also explored whether medium- and far-distance faces were accurately calibrated in two one-samples *t*-tests (also exploratory in nature). For faces encoded at the medium simulated distance, calibration was only marginally different from .90 (the bottom threshold for this bin), $t(85) = 1.70$, $p = .09$, $d = 0.18$ but was significantly and substantially different from .90 for the faces encoded at the far simulated distance, $t(139) = 6.41$, $p < .001$, $d = 0.54$. For faces that were encoded at a near simulated distance, this comparison was not significant, $t(286) = 0.91$, $p = .36$, $d = 0.05$. This provides additional evidence that participants were well calibrated when faces were presented clearly, that participants were perhaps slightly more overconfident for faces that were encoded at a medium-simulated distance and were unequivocally overconfident when the faces had been encoded at a simulated far distance.
